# Smart Control of Nitroxide-Mediated Polymerization Initiators’ Reactivity by pH, Complexation with Metals, and Chemical Transformations

**DOI:** 10.3390/ma12050688

**Published:** 2019-02-26

**Authors:** Mariya Edeleva, Gerard Audran, Sylvain Marque, Elena Bagryanskaya

**Affiliations:** 1 N. N. Vorozhtsov Institute of Organic Chemistry SB RAS, Pr. Lavrentjeva 9, Novosibirsk 630090, Russia; edeleva@nioch.nsc.ru; 2 National Research University—Novosibirsk State University, Novosibirsk 630090, Russia; 3 Aix Marseille Univ, CNRS, ICR, UMR 7273, case 551, Avenue Escadrille Normandie-Niemen, 13397 Marseille CEDEX 20, France; g.audran@univ-amu.fr (G.A.); sylvain.marque@univ-amu.fr (S.M.)

**Keywords:** nitroxide mediated polymerization, alkoxyamine, tunable rate constants, protonation, complexation

## Abstract

Because alkoxyamines are employed in a number of important applications, such as nitroxide-mediated polymerization, radical chemistry, redox chemistry, and catalysis, research into their reactivity is especially important. Typically, the rate of alkoxyamine homolysis is strongly dependent on temperature. Nonetheless, thermal regulation of such reactions is not always optimal. This review describes various ways to reversibly change the rate of C–ON bond homolysis of alkoxyamines at constant temperature. The major methods influencing C–ON bond homolysis without alteration of temperature are protonation of functional groups in an alkoxyamine, formation of metal–alkoxyamine complexes, and chemical transformation of alkoxyamines. Depending on the structure of an alkoxyamine, these approaches can have a significant effect on the homolysis rate constant, by a factor of up to 30, and can shorten the half-lifetime from days to seconds. These methods open new prospects for the application of alkoxyamines in biology and increase the safety of (and control over) the nitroxide-mediated polymerization method.

## 1. Introduction

### Nitroxide-Mediated Polymerization. The General Concept of “Smart Alkoxyamines”

Alkoxyamines are adducts of stable nitroxides with C-centered radicals. Invented as an initiator for nitroxide-mediated polymerization (NMP), [1,2] nowadays alkoxyamines find a wide range of applications including tin-free organic radical chemistry, [3] as initiators for radical cyclization, [4] radical addition reactions, [5,6] creation of self-healing polymers, [7] optoelectronic materials, [8] and encoding systems, [9] and in biomedicine, as theranostic agents. [10] The majority of applications involve the ability of alkoxyamines to undergo C–ON bond homolysis after heating, thus releasing nitroxide and an alkyl radical. Therefore, factors affecting stability of the C–ON bond are important. An example of such significance is given by Chauvin et al., [11] who studied the influence of the initiation rate on the regime of NMP. As they demonstrated, a fast initiation rate guarantees rapid achievement of the controlled mode of polymerization.

The principal application of alkoxyamines is initiation of NMP because the equilibrium between unimolecular and macromolecular alkoxyamines and nitroxides and C-centered radicals is the main process in this type of polymerization. The mechanism of NMP is based on the phenomenon known as the “persistent radical effect” discovered by Fischer and Fukuda. Due to this effect, at the very first steps of polymerization, a small excess of nitroxides over C-centered radicals is formed. This makes the reaction of the nitroxide and polymer radical recombination predominant and reduces the impact of the cross-coupling reaction. As a result, the majority of polymer chains contain the alkoxyamine function as an end group, thus making the reinitiation reaction possible so that the polymer chains become “living”. Another important feature of NMP is a direct linear relation between monomer conversion and the molecular weight of the polymer obtained. Along with low toxicity of nitroxides, the two abovementioned key properties make NMP attractive for the synthesis of polymers with complex structures and composition. The detailed description of the mechanism and kinetics of controlled radical polymerization is well described in the following papers and reviews: [12,13,14,15,16].

**Figure 1:**
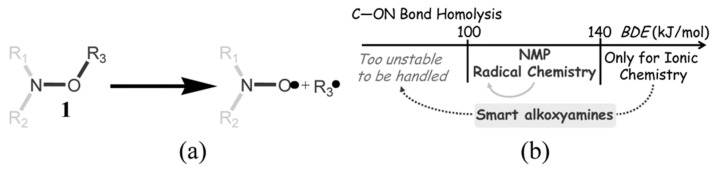
(**a**) The scheme of alkoxyamine hemolysis; (**b**) An outline of a smart alkoxyamine. Reproduced/Adapted from Ref. [18] with permission from The Royal Society of Chemistry.

The reaction of C–ON bond homolysis is unimolecular (Figure 1a); thus, for the sake of simplicity, it is customary to compare the activation energies of this reaction among different types of alkoxyamines. The vibration factor was determined and turned out to be 2.4 × 10^14^ s^−1^ [17] Depending on the structure, alkoxyamines can be either very labile with *E*_a_ below 100 kJ/mol and half-lifetime in the range of minutes or stable with *E*_a_ more than 140 kJ/mol, in which case, alkoxyamines can hardly undergo homolysis even at elevated temperatures. The optimal range of activation energies for alkoxyamines suitable for NMP is between 100 and 140 kJ/mol [10] 

Because alkoxyamines are employed as an initiator for polymerization, they should fulfill all the safety requirements for this type of compounds [19]. On the other hand, “fast” alkoxyamines perform better in NMP. Consequently, an “ideal” alkoxyamine must possess two antagonistic properties, that is be safe to handle and decompose fast when it is needed as a polymerization initiator. This review describes the concept of so-called smart alkoxyamines, which can change their reactivity after an external trigger by converting from “stable and safe” to “fast and effective” (Figure 1b) [18]. 

**Figure 2:**
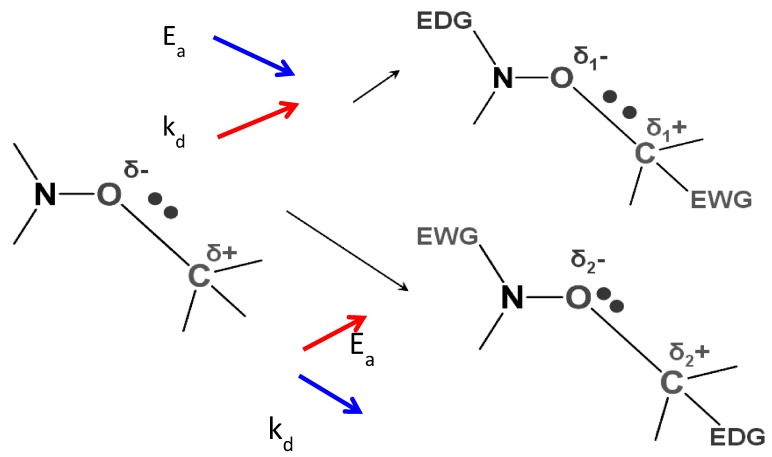
The influence of introduction of electron-withdrawing groups and electron-donating groups into the alkyl and nitroxyl part of an alkoxyamine on the polarity of the C–ON bond and thus the rate of homolysis.

There are several factors that influence reactivity of alkoxyamines. Among them, electron-withdrawing and electron-donating properties of substituents are of special importance in terms of the influence on reactivity of the C–ON bond. Because it is polar with δ^−^ located on the oxygen atom, all electronic effects that reduce polarity of the bond should favor homolysis (Figure 2). That is, if an electron-withdrawing substituent is introduced into the alkyl part, one can expect the decay of homolysis activation energy and vice versa. 

In this subsection, we review possible scenarios related to reversible switching of electron-donating properties of substituents described in the literature. The factors that induce changes in polarity without affecting alkoxyamine substituents are described as well.

## 2. Chemical Activation of C–ON Bond Homolysis

Chemical activation did not attract much interest during the first decade despite the unexpected efficiency of NMP of vinylpyridine under acidic conditions as reported by Fischer et al. in 1999. [20] A decade later, Marx et al. [21] and Mazarin et al. [22] reported a possible occurrence of chemical activation but did not provide experimental evidence. 

**Figure 3:**
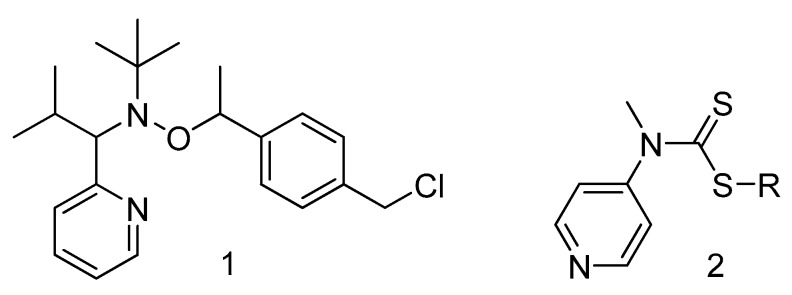
Structure of alkoxyamine 1 and RAFT agent 2 first used for pH-switchable controlled radical polymerization.

Simultaneously, a new concept of pH-switchable agents for reversible addition−fragmentation chain transfer (RAFT) polymerization was introduced (Structure 2 in Figure 3) [23,24]. It was shown that the activation and deactivation parameters of RAFT agents can vary after protonation of functional groups in dithiocarbamates serving as control agents. By means of this effect, the polymerization of so-called more activated monomers and less activated monomers can be performed in a controlled manner with the same initiator or controlling agent. This strategy offers a facile route to the preparation of block-copolymers. A similar concept can be applied to control the reactivity of alkoxyamines.

In this subsection, we will consider the experimental and theoretical evidence of the effect of protonation on C–ON bond homolysis in alkoxyamines. Given that alkoxyamines consist of two parts, there are two opportunities for protonation or deprotonation, which have opposite effects of C–ON bond homolysis, such as the protonation of the alkyl and nitroxyl part. In the text below, we will go through each of these options and consider different influences on reactivity that are promoted after protonation.

### 2.1. Activation by Protonation or Deprotonation

#### 2.1.1. Protonation of the Alkyl Moiety 

**Figure 4:**
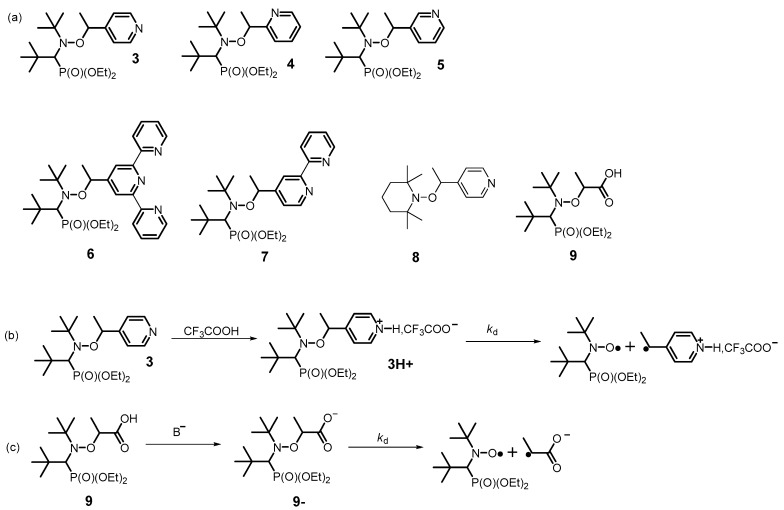
(**a**) Structures of alkoxyamines **3**–**9**; **(b**) Protonation of **3** and (**c**) deprotonation of **9**.

The very first experimental report on the activation of C–ON bond homolysis by protonation of the alkyl moiety (Figure 4) was published by Brémond and Marque [25] in 2011, where they compared activation energies of nonactivated alkoxyamine **3** (*E*_a_ = 123.0 kJ/mol) and its protonated form **3H**^+^ (*E*_a_ = 115.0 kJ/mol). As a result, they observed a 10-fold increase in *k*_d_ after the protonation of **3** (Figure 4).

The same has been observed during protonation of *ortho*-isomer **4 [26]** and *meta*-isomer **5 [27]** though the latter has manifested a smaller effect, as expected (vide infra). It should be noted that when terpyridine or bipyridine is the substituent of alkyl fragments (as in **6** and **7**), the effect of protonation also enhances *k*_d_. Furthermore, protonation has been investigated for alkoxyamine **8**, which is composed of TEMPO as a nitroxyl moiety and ethylpyridine as an alkyl part. The deprotonated alkoxyamine has a threefold lower *k*_d_ than the protonated one does [28]. 

The effect of the alkyl moiety deprotonation was first investigated with alkoxyamine **9**, [29] which contains an alkyl moiety carrying a carboxylic function. The differences in *E*_a_ between carboxylic alkoxyamine **9** and its deprotonated forms carboxylate alkoxyamines **9^−^** are not significant: less than 2 kJ/mol (Figure 4).

#### 2.1.2. Protonation of the Nitroxyl Part 

As mentioned above, the protonation of alkyl and nitroxyl parts has different effects on C–ON bond homolysis. In this subsection, we consider the influence of nitroxyl moiety protonation on *E*_a_ of homolysis.

The study by Edeleva et al. [30] extends the concept of pH-switchable mediators to NMP by employing nitroxides with basic or acidic groups as controlling agents. The nitroxides described there belong to the imidazoline family and are known to be pH sensitive, meaning that the hyperfine-coupling constant depends on protonation of the imidazoline nitrogen. This phenomenon implies that protonation affects electron density on the nitrogen atom. Therefore, the authors expected that protonation would influence electronic properties of the C–ON bond as well.

**Figure 5:**
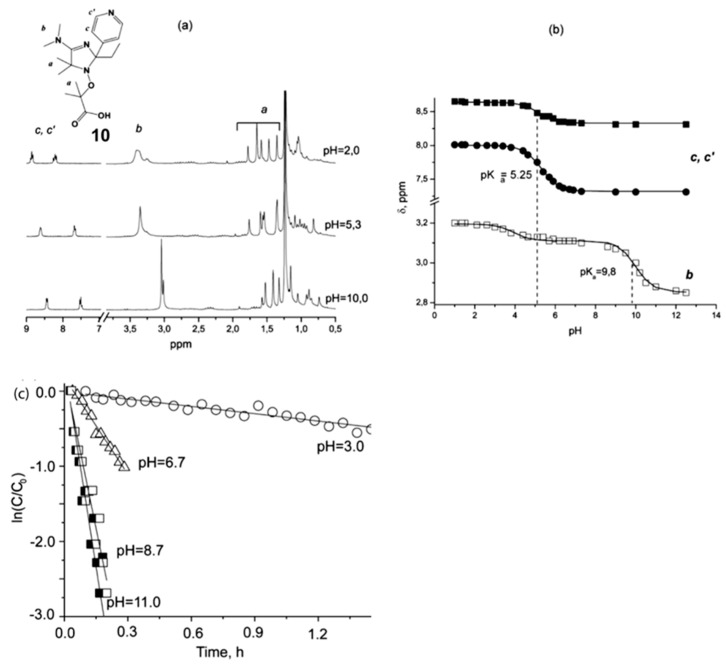
(**a**) ^1^H NMR spectra of **10** (in D_2_O) recorded at solution pH levels 10.0, 5.3, and 2.0 with signal attribution as indicated in the structure; (**b**) Titration curves obtained from signals b (□) and c,c’ (■, ●) (see signal attribution) with a fit, and the values of pK_a_ for pyridine and amidine functions; (**c**) Kinetics of homolysis of alkoxyamine **10** (0.02 M solution) at 368 K as determined by ^1^H NMR in the presence of 40 eq. of ascorbic acid or ascorbate at different pH levels. Adapted with permission from Ref. [30]. Copyright 2011 American Chemical Society.

In the abovementioned article, the first study on the effect of pH on rate constants *k_d_* and *k_c_* was conducted by NMR and EPR spectroscopy. The homolysis rate constants for alkoxyamines **10–13** and rate constants *k*_c_ for recombination of the respective nitroxides with different alkyl radicals were measured at different pH levels. As the first evidence, NMR spectra of alkoxyamines **10–13** in D_2_O revealed pH dependence (see Figure 5 for an example of alkoxyamine **10**). Due to a fast exchange of protonated/deprotonated forms, which results in narrow NMR lines, the authors were able to monitor pH-associated changes for each isomer separately. Using this pH dependence of NMR resonance, the authors could build titration curves for protonable groups (Figure 5b). For alkoxyamine **10** (see Figure 5), *k*_d_ at acidic pH was found to be 15-fold lower than that in a basic medium; this phenomenon is likely due to a synergetic effect of the protonation of both the alkyl and nitroxide moiety. For different diastereomers, the difference in *k_d_* values was within the margin of experimental error. It should be noted that the addition of an acid does not affect *k*_d_ for TEMPO-based alkoxyamines in organic media. 

The influence of protonation on recombination rate constants *k*_c_ has been investigated by laser flash photolysis in the abovementioned article. The observed impact of protonation on *k_c_* is not large and is opposite to the influence on *k_d_*.

Later, Le Du et al. [31] have used a 2,2,5-tri-methyl-4-phenyl-3-azahexane-3-nitroxide type of nitroxide with a pyridyl substituent to investigate the influence of protonation on noncyclic nitroxides. As in the work of Edeleva et al., after protonation, they observed a ~1.9-fold decrease in *k*_d_ at 100 °C.

#### 2.1.3. Theoretical Research

**Figure 6:**
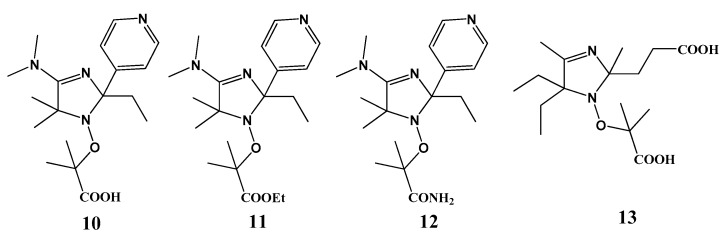
Structures of alkoxyamines **10**–**13**.

To get a deeper insight into the effect of protonation on alkoxyamine homolysis, Parkhomenko et al. [32]. have performed density functional theory (DFT) calculation of Gibbs free energy of the homolysis reaction for alkoxyamines **10**–**13** (Figure 6). In this case, the reactivity was mainly determined by destabilization of the radical products of C−ON bond scission reactions. Those authors observed linear correlations between radical stabilization energies of products of homolysis and calculated Δ_r_^0^G. 

**Figure 7:**
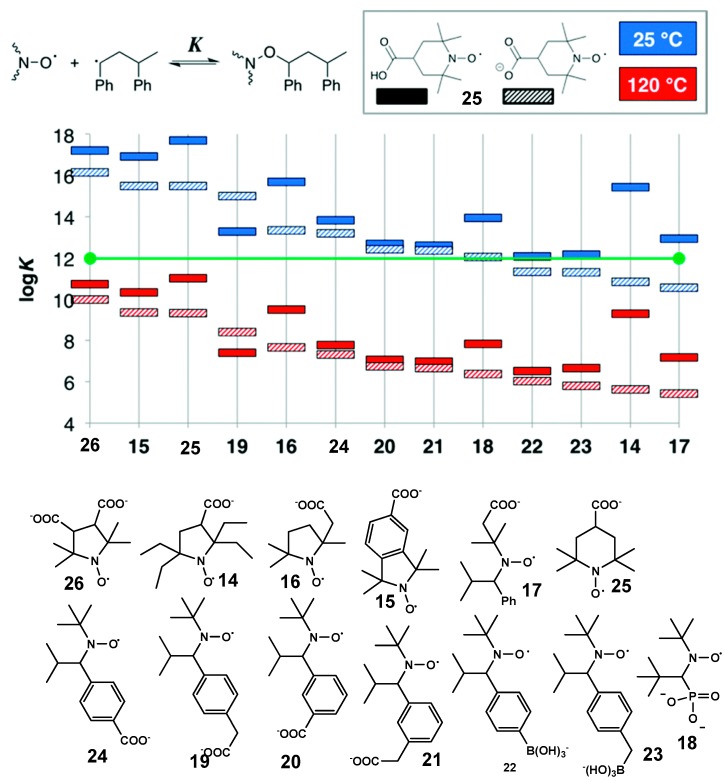
Logarithms of equilibrium constants for NO–C bond homolysis (a combination is defined as the forward reaction, and decomposition as the reverse one) obtained from the bond dissociation free energies for the tested nitroxides with a styryl dimer as a propagating radical, calculated for a bulk styrene solution at 25 and 120 °C. The green line corresponds to log K = 12 (corresponds to the TEMPO-Sty alkoxyamine used as a reference). Structures of only the anionic forms are depicted. Adapted from Ref. [33] with permission from the PCCP Owner Societies.

Later, Gryn’ova et al. [33] theoretically evaluated several known and novel nitroxides with protonable groups as possible mediators of NMP of styrene at room temperature (Figure 7). As a parameter, they calculated equilibrium constant K of the alkoxyamine decomposition reaction at room temperature and at 120 °C for protonated and deprotonated species. It is well known that TEMPO successfully mediates styrene polymerization at 120 °C; hence, homolysis equilibrium constant K for the corresponding alkoxyamine TEMPO–STY was used as a reference. Systems with a lower K are expected to succeed, whereas species with log K > 12 may be too stable to release the propagating radicals at a sufficient rate. They found that nitroxides **14–26** possess suitable homolysis parameters when deprotonated but remain stable when neutral. An acidic derivative of nitroxide SG1 **18** was predicted to be suitable for the NMP of styrene at 60 °C.

It must be noted that at a high temperature, along with the polymerization, the process of depolymerization also becomes well pronounced. The impact of the depolymerization process is described in the review by Tang et al. [34]. 

#### 2.1.4. Nmp Using Initiators with Reactivity Activated by pH

**Figure 8:**
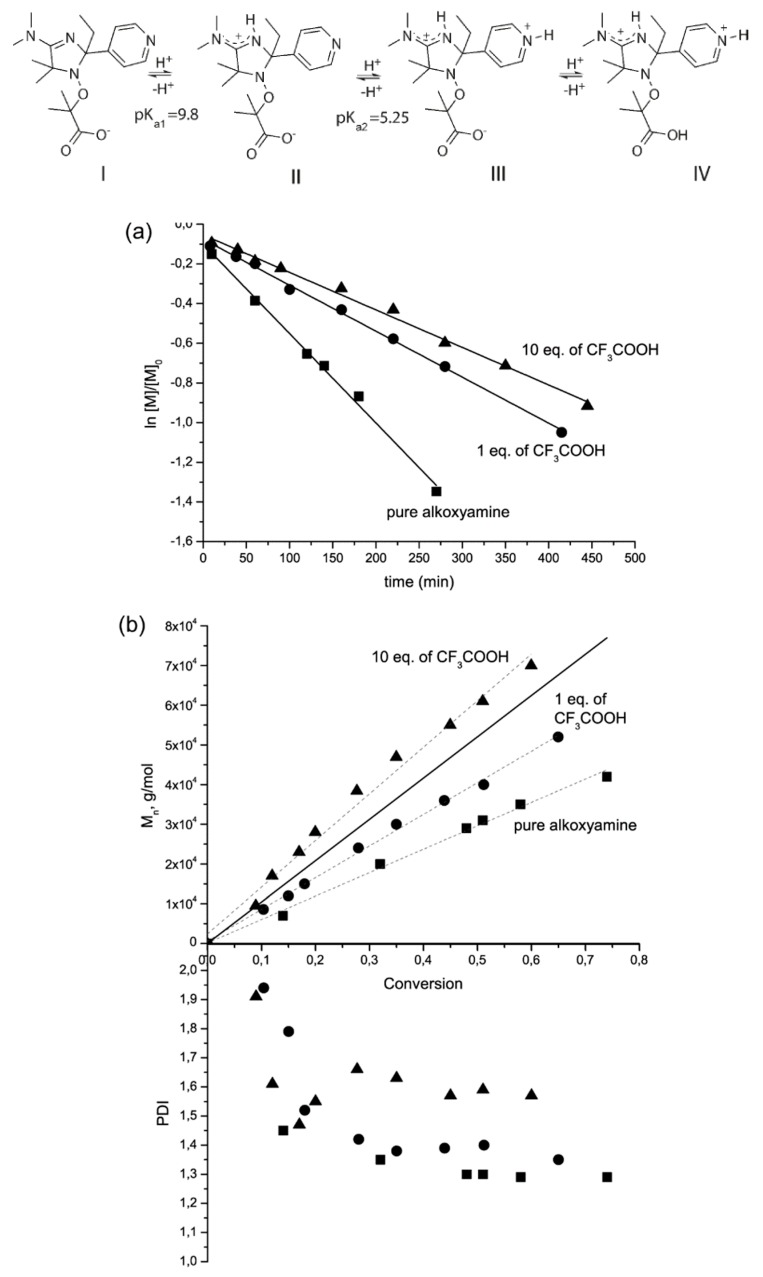
Protonated forms of alkoxyamine **10.** Polymerization of styrene at 140 °C initiated by protonated or deprotonated forms of alkoxyamine **10**. The monomer-to-initiator ratio is 1000/1. (**a**) A kinetics plot for polymerization; lines: a linear fit of the experimental data points; (**b**) Evolution of molecular weight and dispersity. ■: pure alkoxyamine, form II; ●: alkoxyamine in the presence of 1 eq. of CF_3_COOH, form III; ▲: alkoxyamine in the presence of 10 eq. of CF_3_COOH, form IV. The solid line denotes the theoretical M_n_, dashed lines: a linear fit of the experimental data points. Adapted with permission from Ref. [30]. Copyright 2011 American Chemical Society.

The influence of protonation on the type of NMP was first demonstrated by application of imidazoline-based alkoxyamines for the initiation of polymerization of styrene and some water-soluble monomers, such as acrylamide and styrene sulfonate at temperatures below 100 °C. As the first step, those authors performed Fischer’s diagram [35] analysis for polymerization of styrene at 140 °C and acrylamide at 90 °C (Figure 8). This analysis indicates that one can expect controlled polymerization of styrene mediated by deprotonated and monoprotonated forms of alkoxyamine **10**, whereas for acrylamide polymerization, the alkoxyamine should be in a fully protonated form. NMP of styrene initiated by all the forms of the alkoxyamine revealed that the deprotonated form affords a polymer with the polydispersity index (PDI) higher than 1.5. A controlled regime was achieved too for acrylamide polymerization. The livingness of the polymer was verified in reinitiation experiments. In all cases, the increase in molecular weight and the absence of macroinitiator traces were observed by gel permeation chromatography indicating the living character of the polymerization. The importance of this work is also exemplified by the expansion of applications of imidazoline-based nitroxides to aqueous monomers because this type of nitroxides is effective at polymerization of acrylates, styrene, [15,36,37,38,39,40] and even methacrylate. [41,42]

The potential usefulness of protonated or deprotonated forms of SG1-based alkoxyamines in NMP has been tested via polymerization of styrene and styrene sulfonate at 110 and 90 °C. [43] NMP of styrene at 110 °C was successful for both **3** and **3H^+^** (see Figure 4 for structures) judging by linear kinetics, a linear increase in molar masses M_n_ with conversion, and a decrease in the PDI with conversion. NMP of styrene initiated by **3H^+^** (1000 min, 30% conversion, PDI = 1.20) was as good as that initiated by BlocBuilder^TM^ (3900 min, 58% conversion, PDI = 1.23) [11], although due to the decay of the nitroxide under acidic conditions, the polymer’s molecular mass did not change above 30% conversion. For aqueous polymerization of sodium styrene sulfonate, good results were obtained for the deprotonated form of the alkoxyamine despite unfavorable *k*_d_: 70% conversion in 400 min, linear evolution of M_n_, and PDI = 1.4; these characteristics are comparable to those of BlocBuilder^TM^ [43]. 

Le Du et al. [31] have utilized pH-switchable alkoxyamines based on 2,2,5-tri-methyl-4-phenyl-3-azahexane-3-nitroxide (TIPNO) to control the polymerization of styrene and butyl acrylate. For both monomers, a controlled regime of polymerization was observed with narrow polydispersity of the resultant polymer. Meanwhile, for deprotonated forms, the kinetics of monomer conversion were faster than those for the deprotonated ones.

Of note, when an alkoxyamine bears a protonable unit in the alkyl part, the influence of protonation is limited to the initiation step. Therefore, in such cases, protonation is a good way to influence the initiation rate. In the case of protonation of the nitroxyl part, the overall kinetics of polymerization are affected.

### 2.2. Activation by Formation of Metal–Alkoxyamine Complexes

#### 2.2.1. Formation of Metal–Alkoxyamine Complexes: Alkoxyamines with Cu(hfac)_2_, Zn(hfac)_2_, or Tb(hfac)_3_. Structure and Influence on k_d_

Aside from protonation, formation of a metal–alkoxyamine complex is another way to alter the C–ON bond reactivity: by changing the polarity of the bond. A recent series of articles describes the synthesis of such complexes, research into the influence of complexation on C–ON bond homolysis, and application to the NMP of styrene and *n*-butyl acrylate [44,45,46]. 

**Figure 9:**
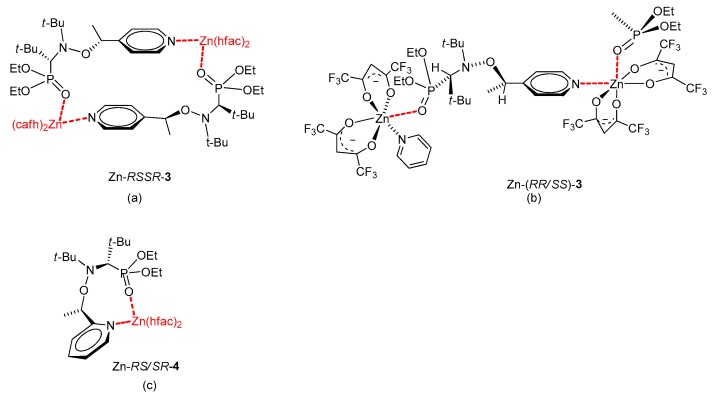
Structures of bis(hexafluoroacetylacetonate) zinc complexes with alkoxyamines **3** and **4** according to X-ray diffraction data: (**a**) the ring-type complex, (**b**) chain-type complex, and (**c**) intramolecular complex. The structure of the complex depends strongly on the diastereomeric configuration of the alkoxyamine.

**Figure 10:**
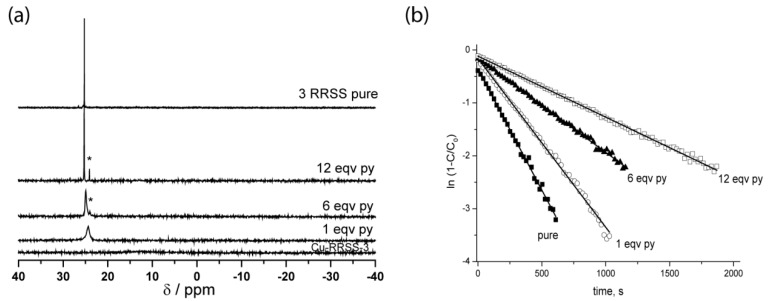
(**a**) Room temperature data on ^31^P NMR spectroscopy at 0, 1, 6, and 12 equivalents of pyridine (from the bottom up) added to Cu-*RSSR*-**3** in C_6_D_6_ (the asterisk denotes free (*RS*/*SR*)-**3** as an impurity) and data on pure 3-RRSS; (**b**) Kinetics of Cu-*RSSR*-**3** decomposition in the presence of 3 eq. of TEMPO after gradual addition of pyridine (py) as a competitor. Adapted from Ref. [44] with permission from The Royal Society of Chemistry.

Coordinately unsaturated copper (II) hexafluoroacetylacetonate [Cu(hfac)_2_], zinc hexafluoroacetylacetonate, and terbium hexafluoroacetylacetonate are the most suitable compounds for the synthesis of such complexes because these salts are strong Lewis acids and yield well-crystallizing coordination compounds. Nevertheless, the interaction of these salts with ligands can form different types of complexes depending on the conditions [47]. This notion has been validated by careful evaluation of the crystal structure of the complexes obtained from the salts and alkoxyamines **3**–**5** based on nitroxide SG1 (Figure 9). The complexes can be intramolecular (M-*RS/SR*-**4**), ring-type (M-*RSSR*-**3**), or chain-type (M-(*RR/SS*)-**3**) when coordinated to Cu or Zn. The structure of Tb-based complexes is completely different. X-ray diffraction analysis has uncovered formation of complexes in which only the nitroxyl part of alkoxyamines is coordinated. These compounds are the first example of coordination between the center cation and the nitroxyl moiety only. The stability of these complexes in solution was checked by NMR spectroscopy. For Cu-based complexes, the ^31^P NMR resonance is strongly shifted relative to the NMR line of a pure alkoxyamine and broadened by a paramagnetic copper ion. Given that Zn is diamagnetic, stability of the complex is indicated only by a downfield shift of the signal. In both cases, pyridine was chosen to decompose the complexes owing to a higher complexation constant with metals. Indeed, for Cu(II) complexes, pyridine has a valuable property in that it reacts quickly (and often irreversibly) [48,49] with the Cu(II) ion to displace weakly bonded ligands. When the concentration of pyridine increased, ^31^P resonance shifted toward the value of the pure alkoxyamine (Figure 10a).

After careful evaluation of the structure of the complexes in solutions, the influence of complexation on homolysis rate constants was studied. It should be pointed out that the structure of the complex determines whether complexation raises or lowers *k*_d_. As an example, for the complex Cu-RSSR-**3**, the authors observed a 10 kJ/mol decrease in *E*_a_. After the addition of various amounts of pyridine, a gradual decrease in *k*_d_ was registered (Figure 10b).

In comparison with protonation, complexation allows for step-by-step alteration of homolysis rate constants; this approach can help to achieve a controlled regime for different monomers.

When Tb-based complexes were studied by ^1^H and ^31^P NMR spectroscopy, the authors observed a strong paramagnetic shift and broadening of signals because of the paramagnetic properties of the Tb(III) cation. After the addition of various competitors, the equilibrium between the complexed and free form of alkoxyamine shifts toward the latter, and the recovery of the NMR signal in spectra can be seen. The addition of pyridine had almost no effect on the shift of the equilibrium because no revival of the signal was observed up to 1000 eq. of the competitor added. When 300 eq. of tetramethylethylenediamine (TMEDA) or bipyridine was added, a broad ^31^P NMR signal was detected. Thus, bipyridine was found to be effective in decomplexation of an alkoxyamine.

#### 2.2.2. NMP of Various Monomers with Initiators in the Form of a Metal–Alkoxyamine Complex 

**Figure 11:**
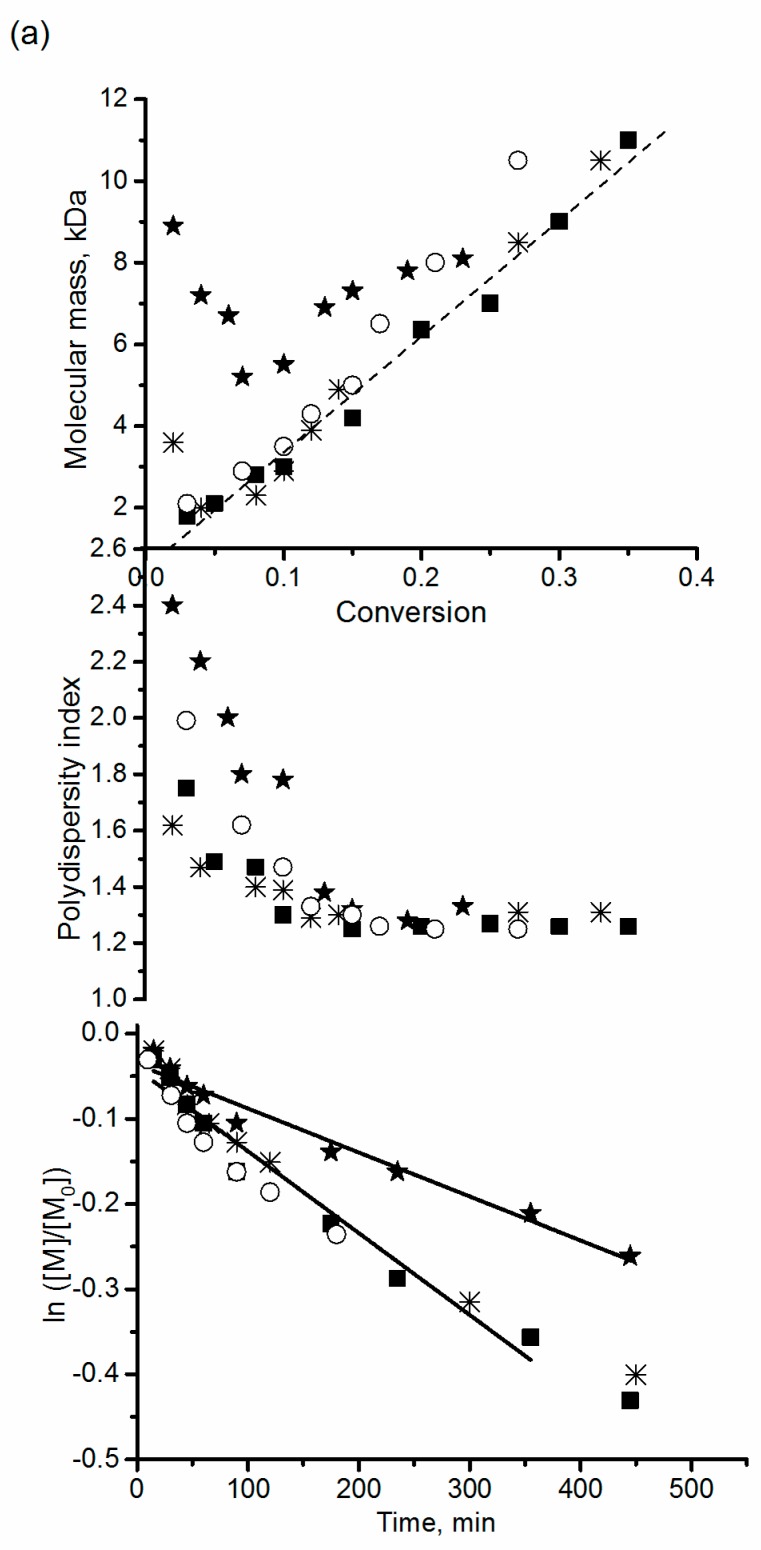
M_n_ versus conversion, PDI vs. conversion, and ln([M]/[M]_0_) vs. time plots for (a) the polymerization of styrene at 90 °C initiated with *RS/SR*-**3** (★), Zn-*RSSR*-**3** (■), Zn-*RSSR*-**3’** (*RS/SR*-**3** + 0.5 eq. Zn(hfac)_2_) (*), or **3** + 10 eq. Zn(hfac)_2_ (○); the monomer to initiator ratio is 250:1.

Polymers doped with metals have found a wide variety of applications. NMP with metal–alkoxyamine initiators means introduction of a straightforward method for preparation of such compounds. The application of alkoxyamine complexes with zinc hexafluoroacetyl acetonate for polymerization of styrene and *n*-Bu-acrylate has been demonstrated by Edeleva et al. [50]. They observed a poorly controlled regime when a free form of alkoxyamine was applied to the NMP of styrene. By contrast, when a presynthesized or in situ–generated complex served as the initiator, a controlled regime was rapidly established. It should be mentioned that NMP initiated by alkoxyamine complexes of a metal cation has all the features of conventional NMP, that is, good control of molar masses, PDI below 1.5, and livingness. Moreover, NMP performed using in situ–generated complexes has the same features as those of the NMP initiated by complexes prepared beforehand, even with a large excess of the metal cation (Figure 11).

### 2.3. Activation via Chemical Transformations 

Thus far, we have considered protonation and formation of complexes as a way to alter the reactivity of alkoxyamines. Other approaches, in particular chemical transformation, can be proposed too. They include reactions with Lewis acids, quaternization of nitrogen, oxidation, and formation of complexes with metal-containing enzymes. In the text below, we present the influence of the abovementioned factors on homolysis rate constants of a series of alkoxyamines.

#### 2.3.1. Lewis Acid and Quaternization

**Figure 12:**
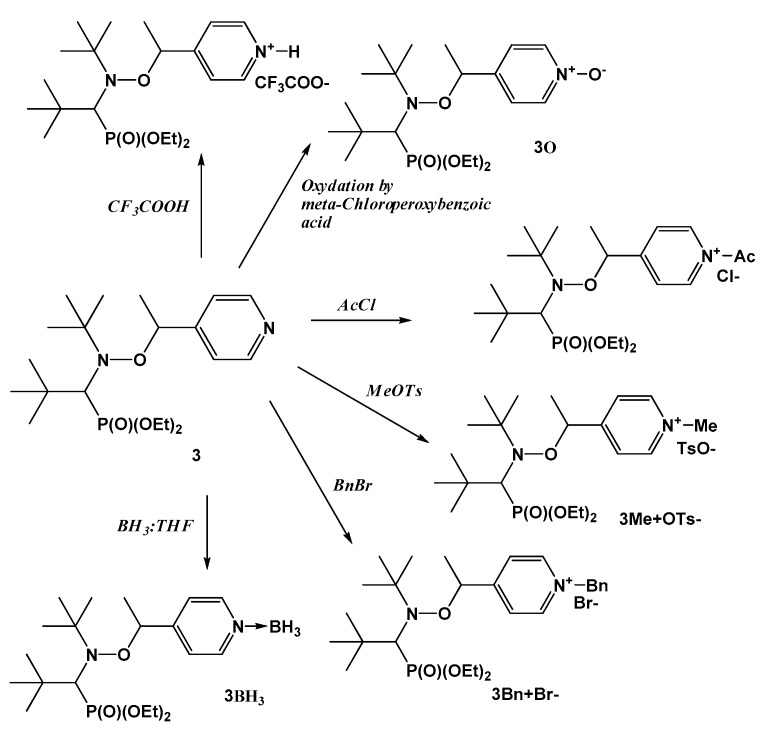
Structures of alkoxyamine **3** derivatives. Adapted with permission from Ref. [51]. Copyright 2012 American Chemical Society.

The effect of coordination of alkoxyamine **3** was investigated with a Lewis acid to afford **3**BH_3_ (Figure 12) [51]. The effect on *k*_d_ was similar to that observed for protonation, that is a 7 kJ/mol difference in activation energies between active and inactive forms. 

Alkoxyamine **3** was also activated by methylation (alkylation), benzylation, and acetylation, with all of them yielding salts with positive charges on the nitrogen atom of the pyridyl ring and the associated counteranion. The differences in *E_a_* were up to 17 kJ/mol. 

The findings reported in the aforementioned articles show the importance of polarity of the alkyl moiety for *k*_d_. Because such modifications change the homolysis activation energy up to 20 kJ/mol, they also generate a substantial gap in half-lifetimes, for example 700 days for the nonactivated form to 5 h for activated ones. Such a difference is important for NMP applications.

#### 2.3.2. Activation by Oxidation

Oxidation of functional groups of an alkoxyamine is another way to alter the polarity. The difference between oxidation and activation by acids or metals is that oxidation is irreversible because it forms neither a salt nor a coordination/dative bond. Furthermore, despite the presence of a positive charge on the nitrogen atom and of the negative charge on the oxygen atom, changes in *k*_d_ are determined by stabilization of the released alkyl radical in sharp contrast to the other types of activation. In the study by Bremond et al. [51], the influence of oxidation was investigated for alkoxyamine **3**. Upon oxidation, it was found to form pyridine-N-oxide derivative **3O** (Figure 12). *E*_a_ is 9 kJ/mol smaller for the latter than for nonoxidized pyridine-based alkoxyamine **3**.

#### 2.3.3. Biological Activation

**Figure 13:**
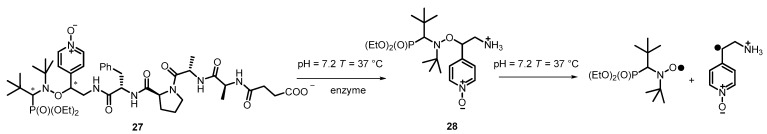
Enzymatic hydrolysis of **27** into **28H**, protonated as **28H^+^** at pH 7.2, and its subsequent spontaneous homolysis into an alkyl radical and nitroxide.

This mode of activation was recently developed by Marque and colleagues for therapeutic applications of alkoxyamines. Thus, alkoxyamine **28** is attached to a peptide to form alkoxyamine **27**, which is specific for chymotrypsin and subtilisin A as enzymes (Figure 13). It was demonstrated that in the presence of one of these enzymes, *k*_d_ of **28** is very similar to *k*_d_ of **28H^+^**, whereas in the presence of porcine pancreatic elastase and of bovine trypsin, the *k*_d_ values are the same as those of pure **27** (Figure 13). Accordingly, alkoxyamine **27** is hydrolyzed by the targeted enzymes into **28**, which is instantaneously protonated into **28H^+^** (p*K*_a_ = 7.97) at pH 7.2. The latter is cleaved faster than the nonenzymatically activated alkoxyamine. As far as we know, this is the first report concerning homolysis of alkoxyamines activated by enzymes even though homolysis is still too slow for biological applications.

#### 2.3.4. Activation of Alkoxyamine Homolysis by 1,3-Dipolar Cycloaddition 

**Figure 14:**
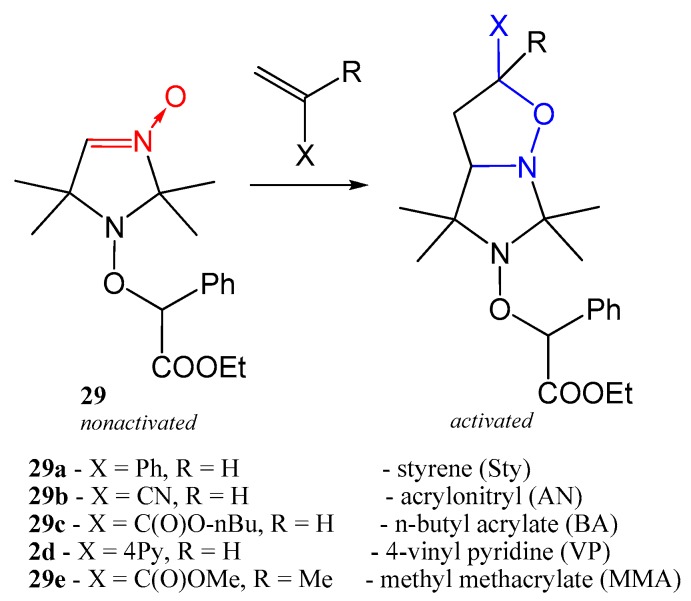
The reaction of 1,3-dipolar cycloaddition of olefin that takes place simultaneously during the initiation of nitroxide-mediated polymerization (NMP).

**Figure 15:**
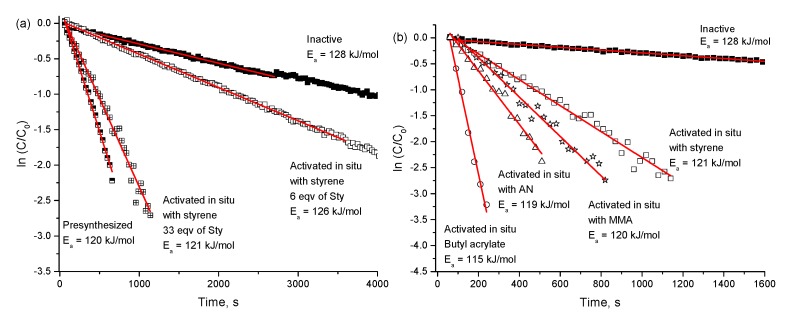
Kinetics of inactive alkoxyamine decomposition under various conditions. (**a**) Black squares: pure inactive alkoxyamine 29, white squares: 6 eq. of styrene, crossed squares: 33 eq. of styrene, half-colored squares: presynthesized with styrene alkoxyamine. (**b**) Black squares: pure inactive alkoxyamine, white squares: 33 eq. of styrene, white stars: 30 eq. of MMA, white triangles: 30 eq. of acrylonitrile, white circles: 35 eq. of butyl acrylate. Red lines: a linear fit of experimental data points. The temperature in all experiments is 373 K. The solvent is C_6_D_4_Cl_2_. Adapted from Ref. [52] with permission from The Royal Society of Chemistry.

An important drawback of the pH control and approaches based on complexation with metals is the necessity to use extraneous additives, which may affect polymer properties. Recently, Edeleva et al. [52] presented a new concept of in situ activation of alkoxyamine homolysis by a 1,3-dipolar cycloaddition reaction with olefins. This concept is rooted in the well-known fact that vinyl monomers involved in NMP usually show high reactivity in cycloaddition reactions. The authors demonstrated the possibility of 1,3-dipolar cycloaddition performed on aldonitrone-containing alkoxyamine **29**. At room temperature, this alkoxyamine is relatively stable due to the electron-withdrawing effect of the nitrone group and is ineffective as an NMP initiator. Under NMP conditions, if the substituent at the fourth position on the imidazoline ring is hydrogen, then it easily reacts with styrene, acrylonitrile, or acrylates to form tricyclic adducts, which have a much higher propensity for C–ON bond homolysis (Figure 14). Figure 15 illustrates the experimental kinetics of nonactivated alkoxyamine **29** decomposition under different conditions and various concentrations of the monomer. The difference between the activation energy of alkoxyamine hemolysis in the nonactivated and activated state is ~9 kJ/mol for styrene and 13 kJ/mol for butyl acrylate. 

### 2.4. Other Factors that Alter Alkoxyamine Reactivity. The Solvent Effect and Intramolecular Hydrogen Bonds

Among other factors that alter alkoxyamine reactivity is the medium. Polarity of the solvent and its ability to favor or suppress formation of hydrogen bonds can have a major influence on *k*_d_. In this subsection, we review the influence of the solvent and formation of IHBs on the homolysis of alkoxyamines. By themselves, they cannot be regarded as factors that alter *k*_d_, but one should keep them in mind when planning NMP and synthetic experiments owing to their huge influence. 

The effects of the solvent on a homolysis reaction can be classified into the following types: (1) a basic solvent effect, which is mediated by polarity of the solvent and its ability to form H-bonds; (2) the solvent effect related to dissociation of a salt; and (3) the solvent effect related to its ability to cleave H-bonds. 

#### 2.4.1. The Basic Solvent Effect

**Figure 16:**
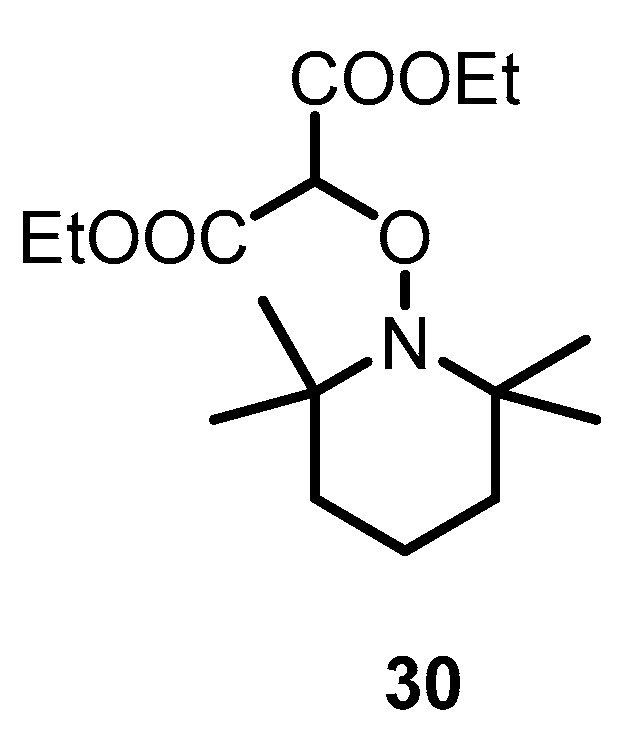
Structure of alkoxyamine **30**.

Solvent effects on alkoxyamines have been investigated during the last three decades [53,54], and it is widely accepted that these effects on alkoxyamines are weak [54], even for activated alkoxyamines [26,55,56,57]. Nonetheless, very recently [28], a 1500-fold increase in *k*_d_ was observed for alkoxyamine **30** after a change of the solvent from *t-*BuPh to a mixture of water and methanol (Figure 16). 

#### 2.4.2. The Influence of the Counteranion

**Figure 17:**
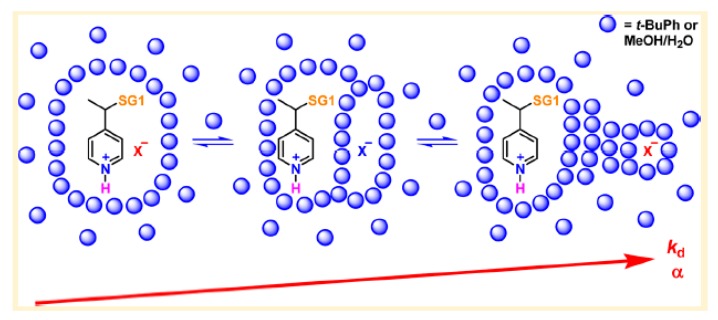
Cartoon representation of the impact of a solvent on an intimate ion pair. Reprinted with permission from Ref. [51]. Copyright 2013 American Chemical Society.

Bremond et al. [51] have observed a clear-cut difference in activation between **3Me^+^TsO^−^** and **3Bn^+^Br^−^** even though the methyl and benzyl groups are both alkylating agents (Figure 17). Nonetheless, their respective counteranions are tosylate and bromide, respectively. Therefore, by means of alkoxyamine **3,** protonation by different acids was investigated in *t-*BuPh and in water–MeOH as a solvent. It was proved that *k*_d_ depends on the counteranions, for example, there is a fourfold increase in *k*_d_ from CF_3_COOH to camphorsulfonic acid in *t*-BuPh, whereas a twofold diminution is observed in water–MeOH [26]. This difference is ascribed to the presence of an intimate ion pair (low *k*_d_) and a solvent-separated ion pair (high *k*_d_) depending on the ability of the solvent to stabilize the anion; this ability roughly correlates with the H-bond donor properties of the solvent α, that is *k*_d_ increases with α. Here, α is an Abraham’s parameter that estimates H-bond donor properties of a solvent [58]. 

#### 2.4.3. IHBs

**Figure 18:**
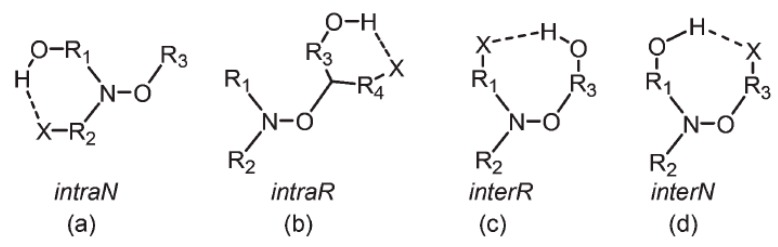

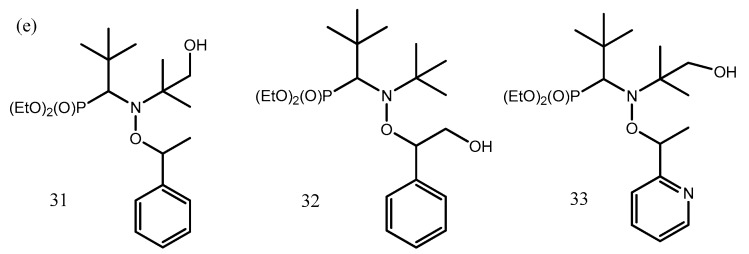
Various types of intramolecular hydrogen bonds (IHBs): (**a**) within the nitroxyl moiety (*intraN*); (**b**) within the alkyl part (*intraR*); (**c**) from an alkyl to nitroxyl part (*interR*); and (**d**) from a nitroxyl to alkyl part (*interN*). Dotted lines represent an IHB. (**e**) Structures of alkoxyamines **31**–**33**.

Despite the weakness of a hydrogen-bonding interaction, it can influence the homolysis of alkoxyamines. If we take into account the structure of alkoxyamines, then four types of IHB are possible (Figure 18): [59] (*a*) *intraN* for IHB in a nitroxyl moiety, (*b*) *intraR* for IHB in an alkyl part, (*c*) *interR* for IHB between nitroxyl and alkyl moieties, the latter carrying the H-donor group, and (*d*) *interN* for IHB between alkyl and nitroxyl parts, the latter carrying the H-donor group. The solvent effect reported in such cases is not always substantial because several factors are involved and sometimes play antagonistic roles, in particular: (i) stabilization of a transition state vs. starting materials by the solvent, (ii) conformational changes suppressing IHB, or (iii) changes in steric hindrance suppressing IHB. In general, suppression of *intraN* IHB by changing the solvent from *t*-BuPh to a water–MeOH mixture slightly decreases *k*_d_ as seen with **31**. On the other hand, suppressing *interN* or *interR* IHB as observed with **32** and **33**, respectively, affords a two- to fivefold increase in *k*_d_ for their *RS*/*SR* diastereoisomers. 

### 2.5. Photochemical Activation of Alkoxyamine Homolysis

Along with thermal homolysis, UV/Vis irradiation can have a large effect on the rate of alkoxyamine C–ON bond cleavage. Photopolymerization represents a rapidly growing field of research because it enables fast polymerization in thin films and raises energy efficiency of the process. A number of papers and reviews describe this important field [60,61,62,63,64,65,66,67]. We will provide only a brief introduction into this field.

The type of NMP that includes photochemical initiation, that is NMP2, involves a chromophoric group directly linked to the aminoxyl function of a nitroxide. As proved by Guillaneuf at al. [61], this type of alkoxyamines undergoes singlet state cleavage and (to a minor extent) possible triplet state cleavage. The abovementioned authors used TEMPO-based alkoxyamines and nitroxide to control the polymerization of *n*-butyl acrylate and observed linear growth of the molecular weight of the polymer.

Another advance in this field is presented in the work of Morris et al. [62], where they studied application of chromophoric-substituted alkoxyamines as a dual initiator in UV-initiated and thermally initiated polymerization for preparation of block-copolymers. Huix-Rotllant et al. [63] performed quantum-chemical calculation to identify the factors that affect the excitation energy transferred from the chromophore to the alkoxyamine moiety. It must be emphasized that photochemical initiation is being developed not only for alkoxyamines but also for other types of polymerization. A number of reviews, papers, and books give a good overview of this field [60,64,65,66,67].

## 3. Conclusions

This review describes the ways to influence the reactivity of alkoxyamines by external chemical stimuli. The rate of C–ON bond homolysis in alkoxyamines is a critical parameter, because for NMP, this parameter can help to achieve a controlled regime of polymerization, whereas for theranostics, rapid homolysis at a low temperature is the key requirement. The factors that change the C–ON bond homolysis rate are temperature, stability of an alkyl and nitroxyl radical released, and polarity of the C–ON bond. The first factor is the most important but also alters the rates of monomer addition and chain termination in NMP. Furthermore, for theranostics, temperature cannot be increased above 37 °C. Radical stabilization is determined by both steric hindrance of the radical center and delocalization of electron density, which are the properties of substituents. The electron-withdrawing or electron-donating characteristics of the latter also affect the polarity of the C–ON bond and thereby control the homolysis rate constant. Consequently, changing these factors can enhance the C–ON bond homolysis rate. Protonation of alkoxyamines and formation of alkoxyamines with metal complexes are new and promising approaches to changing the rate of alkoxyamine homolysis via alteration of electronic properties of the substituents. Protonation of the alkyl part of an alkoxyamine and deprotonation of the nitroxyl part increase the homolysis rate and vice versa. Complexation with metals exerts a similar action.

The possibility of in situ activation is the greatest advantage of both methods. In the case of too slow alkoxyamine homolysis for NMP with a deactivated form of an alkoxyamine, it can be activated by simple addition of an acid or base prior to polymerization. Furthermore, activation by protonation is reversible. If slow initiation is necessary, it can be attained via a change in the acidity of the medium.

Activation with metal ions has many advantages as well. First, it offers additional flexibility in the alteration of kinetic parameters to achieve a controlled regime. Second, this approach does not require additional synthesis because the complex could be obtained in situ. Furthermore, it opens an easy route to metal-polymer complexes that have many valuable properties. Additionally, metal complexes of alkoxyamines can act as orthogonal initiators thus facilitating the synthesis of block-copolymers by different mechanisms.

Nevertheless, various types of activation of alkoxyamines can have some negative effects on the polymerization. Activation by complexation leads to contamination of the polymer with metals, whereas acidity in some cases can affect the stability of nitroxides. Therefore, in each specific case, one should consider both positive and negative consequences of the activation methods.

In our opinion, the most fascinating approach to enhancement of the homolysis rate of an alkoxyamine is the one that involves a 1,3-dipolar cycloaddition reaction of an aldonitrone-substituted alkoxyamine with a monomer. It has been demonstrated that due to this reaction, the half-lifetime of an alkoxyamine shortens from days to minutes. Furthermore, this procedure does not necessitate any additives because only an alkoxyamine and the monomer react, and therefore the final polymer is not contaminated. We consider this approach the most attractive for the industry.

Activation methods lead to both greater versatility in the synthesis of block-copolymers and an increase in NMP energy efficiency, making them more appealing to the industry and academia.
